# Adenosine perfusion cardiac magnetic resonance imaging for risk stratification in patients with stable coronary artery disease - a long-term prospective study in a large consecutive cohort

**DOI:** 10.1186/1532-429X-15-S1-P262

**Published:** 2013-01-30

**Authors:** Peter Bernhardt, Patricia Dewes, Thomas Walcher, Wolfgang Rottbauer, Dominik Buckert

**Affiliations:** 1 University of Ulm, Ulm, Germany

## Background

Adenosine perfusion cardiac magnetic resonance imaging (CMR) has been proven to be useful for short-term prognosis in patients with stable coronary artery disease. Most of the available studies so far only included specific subsets of patients and defined rather soft clinical endpoints. Thus, it remains still uncertain, whether CMR can be used as prognostic tool concerning long-term follow up and the occurrence of major clinical endpoints such as cardiac death or myocardial infarction. Aim of our prospective study was to evaluate the incremental prognostic value of adenosine CMR over other conventional risk factors in a large, consecutive and thus unselected population of patients with stable angina pectoris.

## Methods

Consecutive patients who were referred for adenosine perfusion CMR and who showed symptoms concordant with stable angina pectoris were enrolled unless they exhibited the predefined exclusion criteria. Myocardial perfusion was assessed on a 1.5 T whole-body CMR-scanner by a combination of adenosine stress perfusion and late gadolinium enhancement (LGE). Reversible perfusion deficit was defined as myocardial hypoperfusion during adenosine in the absence of LGE. Primary endpoint was defined as cardiac death, non-fatal myocardial infarction and stroke.

## Results

We were enrolled 1,229 consecutive low to high risk patients. Mean follow-up period was 4.2 ± 2.1 years. In this time, 88 primary endpoints could be observed. The occurrence of cardiac deaths (p < 0.0001) and non-fatal myocardial infarction (p < 0.001) was significantly higher in patients that exhibited a reversible perfusion deficit than in patients without. Performing a multivariate analysis, we could demonstrate that the presence of a reversible perfusion deficit was the strongest independent risk factor for an adverse event with a 3-fold increased risk (Hazard ratio 2.99). In contrast, the absence of a perfusion deficit goes along with a high negative predictive value (95.6 % during the observation period).

## Conclusions

Adenosine perfusion CMR qualifies as a good risk stratification tool with a high long-term prognostic value in patients with stable coronary heart disease. The prognostic information that can be gained is incremental over other conventional risk factors.

## Funding

None

